# Effects of different drugs in combination with PKP/PVP on postoperative pain in patients with osteoporotic compression fractures: a network meta-analysis

**DOI:** 10.3389/fsurg.2024.1349351

**Published:** 2024-07-23

**Authors:** Yiguang Bai, Qiaoling Chen, RouMei Wang, Rui Huang

**Affiliations:** ^1^ Department of Orthopaedics, Nanchong Central Hospital, The Second Clinical Institute of North Sichuan Medical College, Nanchong, China; ^2^ Department of Oncology, Nanchong Central Hospital, The Second Clinical Institute of North Sichuan Medical College, Nanchong, China; ^3^ Department of Medical Ultrasound, The First Affiliated Hospital of Guangxi Medical University, Nanning, China; ^4^ College of Public Hygiene of Guangxi Medical University, Nanning, Guangxi, China

**Keywords:** osteoporosis, osteoporotic fracture, PKP, PVP, network meta-analysis

## Abstract

**Objective:**

This study was designed to evaluate the postoperative pain effect and clinical efficacy of different drugs combined with PKP or PVP in treating osteoporotic vertebral compression fractures (OVCFs) through a systematic review and network meta-analysis.

**Methods:**

We searched five electronic databases, namely, MEDLINE (PubMed), EMBASE, Web of Science, Google Scholar, and the Cochrane Central Register of Controlled Trials online, for the treatment of OVCFs through March 2023 with keywords zoledronic acid (ZOL), teriparatide (TPTD or PTH 1-34), and calcitonin (CT) combined with PKP/PVP. The visual analog scale (VAS) and Oswestry Disability Index (ODI) were the primary outcomes of the network meta-analysis, and the secondary outcome was the diagnostic marker bone mineral density (BMD).

**Results:**

Eighteen studies involving 2,374 patients were included in this study. The network meta-analysis revealed that, in terms of reducing VAS scores, compared with PVP surgery alone, PVP combined with TPTD was most likely to be the treatment associated with the greatest pain relief [MD = −4.99, 95% CI = (−7.45, −2.52)]. In terms of reducing the ODI dysfunction score, compared with PKP combined with Cal, PKP combined with ZOL had the highest probability of being the best treatment option [MD = −9.11, 95% CI = (−14.27, −3.95)]. In terms of protecting against bone density loss, compared with PKP surgery alone, treatment with PKP combined with ZOL had the best effect [MD = 0.39, 95% CI = (0.13,0.65)].

**Conclusions:**

Based on the network meta-analysis and SUCRA rankings, this study concluded that adding teriparatide has the advantage of reducing VAS pain scores compared with PVP alone and that adding zoledronate is a more effective treatment for reducing ODI scores compared with PKP combined with Cal and preserving BMD compared with PKP alone. However, additional high-quality studies are needed to verify our findings.

**Systematic Review Registration:**

https://www.crd.york.ac.uk/PROSPERO/display_record.php?RecordID=358445, identifier CRD42022358445.

## 1 Introduction

Osteoporosis (OP) is a systemic metabolic bone disease characterized by a decrease in bone mineral density (BMD) and mass (1). Osteoporotic vertebral compression fracture (OVCF) is one of the most common and severe complications of OP. According to the International Osteoporosis Foundation (IOF) report, approximately 1/3 of women and 1/5 of men over 50 will suffer from OVCFs (2). Some OP patients with severe bone loss have increased bone fragility, and minor impacts, lifting heavy objects, or even simple sneezing can cause fractures (3). OVCFs often manifest as acute and chronic low back pain, radiating pain, and kyphoscoliosis (4–6) and can lead to disability in patients, affecting not only the quality of life and longevity but also threatening life in severe cases (7, 8). In the context of the aging of the global population, osteoporosis-associated fractures are considered to contribute important economic challenges to worldwide health systems. This highlights the practical importance of investigating therapeutic strategies for osteoporotic fractures (9, 10).

Traditional conservative treatment for OVCFs has not been effective at improving the pain symptoms of patients and may also lead to continued loss of bone mass and increased risk of refracture (11). Today, percutaneous kyphoplasty (PKP) and percutaneous vertebroplasty (PVP) are effective spinal treatment methods that have the advantages of stabilizing the vertebral body structure, relieving fracture pain, reducing disability and accelerating recovery (11, 12). PVP surgery can strengthen the vertebral body by the injection of an appropriate amount of bone cement into the vertebral body. However, this approach has the disadvantage of causing kyphotic deformity of the vertebral body. PKP surgery can compensate for this defect and, at the same time, can relieve fracture pain in the short term. Six months after PKP, most patients may experience pain symptoms again (13). Therefore, postoperative adjuvant anti-osteoporosis drugs may be an effective option for improving patient pain. The identification of drugs that can effectively treat osteoporosis and relieve pain in patients has become an urgent need.

Zoledronic acid (ZOL), teriparatide (TPTD or PTH 1-34), and calcitonin (CT) are clinical drugs for the treatment of OP. ZOL can selectively inhibit the activity of osteoclasts by inhibiting farnesyl pyrophosphate synthase (FPPS) in the mevalonate pathway (14), reducing the ability of osteoclasts to destroy bone tissue and maintain bone mass. Moreover, the benzimidazole heterocyclic structure contained in ZOL endows the drug with a stronger affinity for the bone surface than for the other materials (15), and ZOL is the first-line drug for treating OP (16). TPTD, also known as recombinant human parathyroid hormone 1-34, is composed of the first 34 amino acid fragments of the parathyroid hormone molecule and can activate bone lining cells, promote the maturation and differentiation of osteoblasts, and inhibit the apoptosis of osteoblasts—exemplifying drugs that promote bone formation (17, 18). Peichl et al. conducted a randomized, double-blind, controlled trial of fractures at the pubic bone or pubic symphysis in postmenopausal women. They found that at week 8, patients in the TPTD-treated group healed well and had significantly less pain than patients in the control group (19). CT is a linear polypeptide hormone that contains 32 amino acids and participates in bone calcium metabolism. It can inhibit bone resorption and relieve bone pain. In addition to treating osteoporosis, it can also be used to treat other metabolic bone diseases (20).

Several studies on the use of PKP/PVP in combination with different drugs for treating OVCFs have been reported. However, the efficacy of these different therapies in relieving pain is inconsistent, and there is a lack of systematic analysis comparing the efficacy of different drug combinations. Therefore, based on the findings of previous studies, the purpose of our meta-analysis was to compare the pain relief effects of different therapies for OVCFs, thereby providing additional reference information for future clinical practice.

## 2 Materials and methods

This was a systematic review and network meta-analysis of long-term intervention trials of PKP/PVP combined with different drugs, and this study was conducted strictly according to the registration protocol in PROSPERO (CRD42022358445) and PRISMA guidelines.

### 2.1 Literature search strategy

Adhering to the PICOS framework, the study included the following: (P) population—individuals with osteoporotic compression fractures; (I) intervention—postvertebroplasty or kyphoplasty (PKP/PVP) surgery with concurrent anti-osteoporosis medication; (C) comparator—patients post-PKP/PVP surgery either untreated with anti-osteoporosis medication or treated with alternative pharmacotherapies; (O) outcomes—evaluation of pain, radiological, and laboratory findings; and (S) study type—both randomized and nonrandomized controlled trials.

To identify relevant studies, we carried out comprehensive systematic searches in five electronic databases: MEDLINE (PubMed), EMBASE, Web of Science, Google Scholar, and the Cochrane Central Register of Controlled Trials. The search encompassed the period from the inception of each database to March 1, 2023. According to the PICOS principle, we used the Boolean operators “OR” and “AND” to connect, and the search keywords were “percutaneous kyphoplasty,” “percutaneous vertebroplasty,” “vertebral compression fracture,” “zoledronic acid,” “teriparatide,” “parathyroid hormone 1-34”, and “calcitonin.” There were no language restrictions.

### 2.2 Inclusion and exclusion criteria

Inclusion criteria:

(1) PKP/PVP combined or not combined with different drug interventions
(2) Patients whose follow-up period was not less than one year or longer
(3) Outcome indicators included at least one of the following: visual analog scale (VAS), ODI, or BMD

Exclusion criteria:

(1) Studies with incomplete or unavailable data
(2) Patients with less than one year of follow-up data (1 month, three months, six months, etc.)
(3) Animal studies, conference abstracts, case reports, protocols, correspondences, meta-analyses, and other articles

### 2.3 Study selection and data extraction

**Figure 1:**
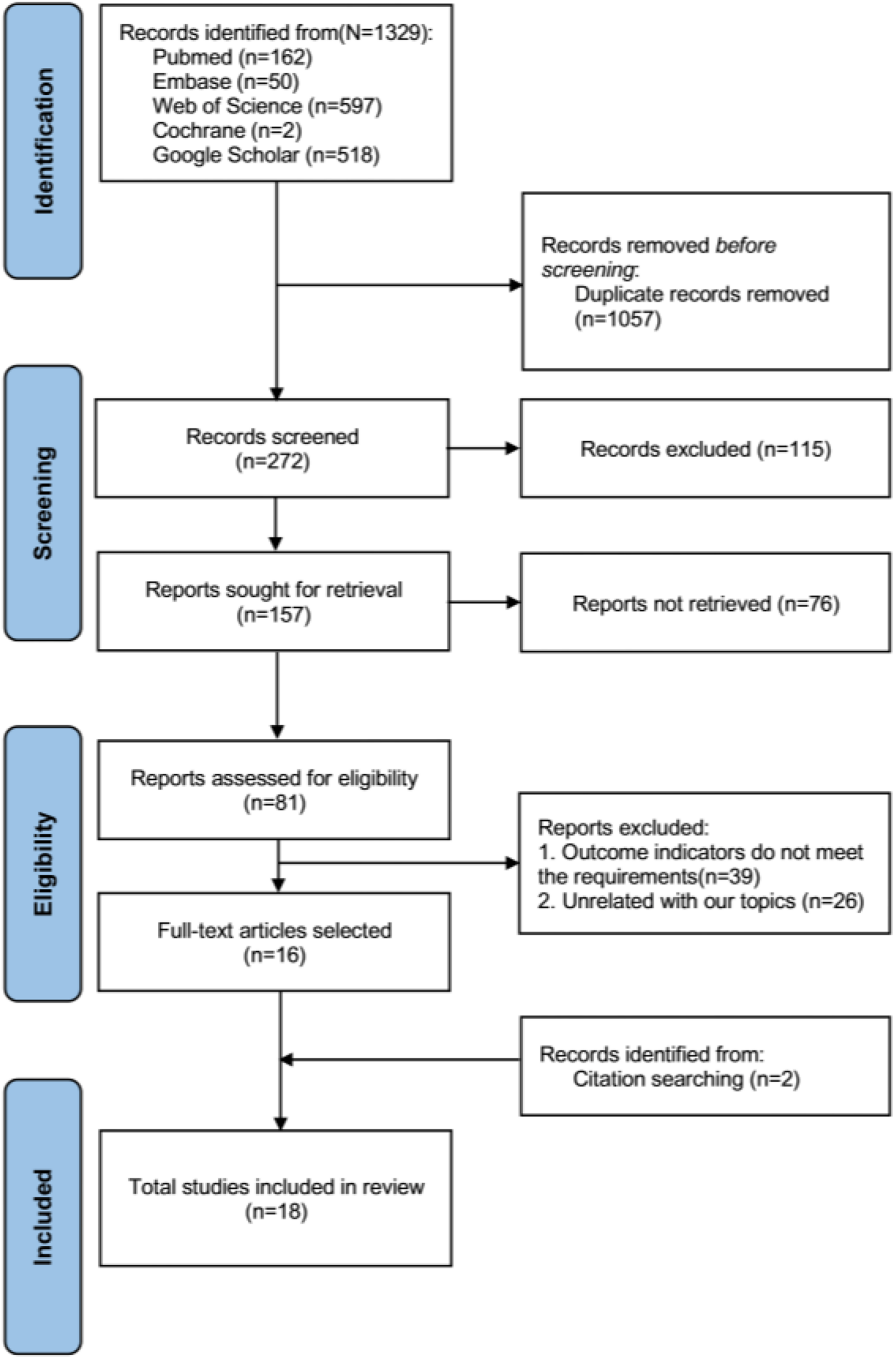
Flow chart of the literature screening process.

The literature search records were systematically managed using EndNote 20 software. The selection process encompassed three distinct phases. During the initial phase, three independent reviewers conducted a preliminary screening of the articles based on their titles; those articles warranting further consideration were retained for abstract review. In the second phase, the initially selected articles underwent abstract review by two independent reviewers to assess their eligibility. Discrepancies in opinion were reconciled through deliberative discussions between the reviewers and, if necessary, in consultation with an additional member of the review team. In the final phase, the same pair of reviewers rigorously examined the full texts of the remaining articles, applying the preestablished inclusion criteria. Any persistent disagreements during this conclusive phase were resolved through comprehensive discussions with the broader review team. The following data were extracted from the included studies: (1) author, (2) country, (3) year of publication, (4) sample size, (5) sex, (6) age, (7) intervention, and (8) study results regarding the VAS score, ODI score and BMD. The primary research outcome was the average change in pain (0–12 months VAS and ODI scores) because pain is one of the essential subjective perceptions of postoperative efficacy. Moreover, evaluating the efficacy of these treatments is an important factor for medical staff. The secondary study outcome was bone density. We reconstructed the numerical data using standard procedures for the graphical VAS and ODI scoring data (21, 22). The flow chart of the literature screening is shown in Figure 1.

### 2.4 Quality assessment and risk of bias assessment

For RCTs, the quality and risk of bias of the included studies were independently and blindly assessed using the Cochrane Collaboration tool, and disagreements during the process were resolved through mutual discussion. The specific evaluation included the generation of a random allocation method, concealment of the allocation scheme, blinding of patients and trial personnel, blinding of outcome assessors, completeness of data, and the presence of selective reporting and other potential biases. According to bias, the assessment criteria and normative standards for the risk of bias in the risk assessment tool classify the research literature into three categories, namely, uncertain risk of bias, low risk of bias and high risk of bias (Supplementary Table S1). For nonrandomized controlled studies, we used the Newcastle–Ottawa Scale (NOS), a systematic review tool for nonrandomized studies, to evaluate three aspects: selection, comparability, and exposure. The total score of each study was 9 points, and the final score was ≥6 points. High-quality documents with a score less than 6 were considered low-quality documents (Supplementary Table S2). In addition, we used graded recommendations, adjudication, development, and evaluation criteria to describe the quality of evidence and strength of the recommendations. According to the GRADE evaluation method, the quality of evidence can be divided into four levels: high, moderate, low, and very low. The initial level of evidence for randomized controlled trials is high, and that for observational studies is low, with five downgrading factors (limitation, imprecision, inconsistency, indirectness, publication bias) and three escalating factors (large effect size, dose‒response relationship, and negative bias) to dynamically evaluate the body of evidence (Tables 2–4). Finally, the approved ethics review agency and ethics review number had to be specified for studies requiring ethical approval.

**Table 1:** Characteristics of the studies included in the network meta-analysis.

Study	Year	Country	No. of patients	Male/female	Age (mean + SD)	Intervention	Outcome^b^ measure
Su (25)	2013	Taiwan, China	65	T: 3:29	T: 77.94 ± 7.44	PVP, TPTD (20 μg)	VAS, ODI, BMD
				C: 3:30	C: 73.12 ± 7.49	Other basic treatments	
Li A (26)	2022	China	90	T: 5:27	T: 69.1 ± 6.9	PKP, TPTD (20 μg)	VAS, ODI, BMD, ABH, MBH, MRABH, MRMBH, KA, DKA, β-CTX, N-MID
				C: 13:45	C: 67.4 ± 5.2	Other basic treatments	
Li B (27)	2020	China	43^a^	T: 3:6	T: 72.3 ± 5.6	PKP, PTH(1-34) (20 μg)	VAS, ODI, BMD, ABH, MBH, KA
				C: 7:15	C: 69.1 ± 4.2	Other basic treatments	
Yuan (28)	2017	China	85	T: 13:30	T:4.23 ± 1.22	PKP, PTH(1-34) (20 μg)	VAS, ODI, BMD, KA
				C: 13:29	C:4.21 ± 1.25	Other basic treatments	
Liu (29)	2017	China	104	T: 13:39	T:67.7 ± 7.6	PKP, ZOL (intravenous drip)	VAS, ODI, BMD, β-CTX, N-MID
				C: 18:34	C:70.9 ± 10.5	Other basic treatments	
Shi (30)	2018	China	95	T: 16:13	T:77.72 ± 5.58	PKP, ZOL (intravenous drip)	VAS, ODI, BMD, VBH, KA, AE
				C: 18:16	C:76.65 ± 4.86	Other basic treatments	
Huang (31)	2019	China	60	T: 10:20	T:76.11 ± 8.30	PKP, ZOL (intravenous drip)	VAS, BMD
				C: 7:23	C:74.36 ± 9.08	Other basic treatments	
Zhang (32)	2019	China	101	T: 0:50	T:64.60 ± 6.70	PKP, ZOL (intravenous drip)	VAS, BMD, N-MID, P1NP, β-CTX, AE
				C: 0:51	C:63.98 ± 7.51	Other basic treatments	
Hu (33)	2020	China	242	T: 49:72	T:62.60 ± 7.20	PVP, ZOL (intravenous drip)	VAS, ODI, BMD, P1NP, β-CTX, AE
				C: 40:81	C:67.45 ± 4.12	Other basic treatments	
Liu (34)	2022	China	238	T: 52:67	T:70.73 ± 5.47	PKP, ZOL (intravenous drip)	VAS, ODI, BMD, N-MID, P1NP, β-CTX, KA, AE
				C: 57:62	C:72.00 ± 5.36	Other basic treatments	
Zhang (35)	2020	China	102	T: 28:26	T:74.07 ± 6.42	PKP, ZOL (intravenous drip)	VAS, BMD, KA, AE, TRACP, CTX
				C: 26:22	C:73.23 ± 7.31	Other basic treatments	
Hao (42)	2021	China	291	T: 99^c^	T:71.48 ± 7.56	PVP, TPTD, ZOL	VAS, EQ-5D
				C: 192^c^	C:70.93 ± 6.81	Other basic treatments	
Dang (36)	2019	China	84	T: 10:30	T: 73.23 ± 4.34	PKP, Cal	VAS, ODI, BMD
				C: 12:32	C: 72.98 ± 4.67	Other basic treatments	
Hao (37)	2018	China	68	T: 12:19	T:69.35 ± 8.86	PKP, Cal	VAS, ODI, BMD, ABH
				C: 10:27	C:70.84 ± 8.45	Other basic treatments	
Zhong (38)	2021	China	60	T: 17:13	T: 63.6 ± 2.3	PVP, Cal	VAS, ODI, BMD
				C: 16:14	C: 62.2 ± 2.1	Other basic treatments	
Wang (39)	2015	China	92	T: 21:25	T: 67.54 ± 7.16	PKP, Cal	VAS, ODI, BMD
				C: 19:27	C: 66.74 ± 6.53	Other basic treatments	
Yi (40)	2020	China	400^c^	T: 81:119	T: 65.13 ± 7.32	PKP, ZOL (intravenous drip)	VAS, ODI, ADL, KA, BMD, BALP, BGP, β-CTX, TP1NP
				C: 82:118	C: 64.61 ± 7.24	Other basic treatments	
Lu (41)	2021	China	154	T: 15:63	T: 68.69 ± 9.31	PKP, ZOL (intravenous drip)	VAS, ODI, BMD, AE, β-CTX, TP1NP
				C: 15:61	C: 70.80 ± 9.11	Other basic treatments	

VAS, visual analog scale; ODI, Oswestry Disability Index; BMD, bone mineral density; PKP, percutaneous kyphoplasty; PVP, percutaneous vertebroplasty; RBP, residual low back pain; ABH, anterior vertebral height; MBH, middle body heights; VBH, vertebral height; MRABH, maintenance rate of anterior body heights; MRMBH, maintenance rate of mid body heights; KA, kyphosis angle; DKA, difference kyphotic angle; β-CTX, beta C-terminal cross-linked telopeptide of type I collagen; N-MID, N-MID osteocalcin; P1NP, procollagen I N-terminal propeptide; TP1NP, total procollagen I N-terminal propeptide; TRACP, tartrate resistant acid phosphatase; BALP, bone specific alkaline phosphatase; BGP, bone morphogenetic protein; EQ-5D, EuroQol Five Dimensions Questionnaire; AE, adverse event; ADL, Activity of Daily Living Scale.

^a^ Only the PKP treatment group and the PKP + PTH(1-34) treatment group were included.

^b^ VAS for 12 months postoperatively in patients with long-term follow-up results.

^c^ The sample size was the number of cases in the study and control groups, excluding group B (PKP combined with ZOL 1 month later).

**Table 2:** Quality of GRADE evidence for postoperative pain VAS scores.

Intervention group	Control group	Limitation	Imprecision	Heterogeneity and inconsistency	Indirectness	Publication bias	Grade
PVP + TPTD	PVP	Downgrade^a^	No downgrade	No downgrade	No downgrade	No downgrade	Moderate
PKP + TPTD	PKP	Downgrade^a^	No downgrade	No downgrade	No downgrade	No downgrade	Moderate
PKP + PTH(1-34)	PKP	Downgrade^a^	No downgrade	Downgrade^c^	No downgrade	No downgrade	Low
PKP + ZOL	PKP	Downgrade^a^	No downgrade	No downgrade	No downgrade	No downgrade	Moderate
PVP + ZOL	PVP	Downgrade^a^	No downgrade	No downgrade	No downgrade	No downgrade	Moderate
PKP + Cal	PKP	Downgrade^a^	No downgrade	No downgrade	No downgrade	No downgrade	Moderate
PVP + TPTD	PVP + ZOL	Downgrade^b^	No downgrade	No downgrade	No downgrade	No downgrade	Moderate
PKP	PVP	Downgrade^a^	No downgrade	No downgrade	No downgrade	No downgrade	Moderate

^a^ >70% contribution from moderate RoB comparisons.

^b^ Because <30% contribution from low RoB comparisons.

^c^ Because node-splitting *p* = 0.013.

**Table 3:** Quality of GRADE evidence for postoperative pain ODI scores.

Intervention group	Control group	Limitation	Imprecision	Heterogeneity and inconsistency	Indirectness	Publication bias	Grade
PKP + TPTD	PKP	Downgrade^a^	No downgrade	No downgrade	No downgrade	No downgrade	Moderate
PKP + PTH(1-34)	PKP	Downgrade^b^	No downgrade	No downgrade	No downgrade	No downgrade	Moderate
PKP + ZOL	PKP	Downgrade^a^	No downgrade	No downgrade	No downgrade	No downgrade	Moderate
PVP + ZOL	PVP	Downgrade^a^	No downgrade	No downgrade	No downgrade	No downgrade	Moderate
PKP + Cal	PKP	Downgrade^a^	Downgrade^c^	No downgrade	No downgrade	No downgrade	Low
PKP	PVP	Downgrade^a^	No downgrade	No downgrade	No downgrade	No downgrade	Moderate

^a^ >70% contribution from moderate RoB comparisons.

^b^ Because <30% contribution from low RoB comparisons.

^c^ Because point estimate >1.0 but lower limit <0.80.

**Table 4:** Quality of GRADE evidence for postoperative pain BMD scores.

Intervention group	Control group	Limitation	Imprecision	Heterogeneity and inconsistency	Indirectness	Publication bias	Grade
PKP + PTH(1-34)	PKP	Downgrade^a^	No downgrade	No downgrade	No downgrade	No downgrade	Moderate
PKP + ZOL	PKP	Downgrade^a^	No downgrade	No downgrade	No downgrade	No downgrade	Moderate
PKP + Cal	PKP	Downgrade^a^	No downgrade	Downgrade^b^	No downgrade	No downgrade	Low
PVP + TPTD	PVP	Downgrade^a^	No downgrade	No downgrade	No downgrade	No downgrade	Moderate
PVP + ZOL	PVP	Downgrade^a^	No downgrade	No downgrade	No downgrade	No downgrade	Moderate

^a^ >70% contribution from moderate RoB comparisons.

^b^ Because point estimate <1.0 but upper limit >1.25.

### 2.5 Data analysis

The data analysis was performed with Stata 15.1 software. In our study, continuous variables are represented as the mean difference (MD), defined as the absolute distinguishing factor between the means of the treatment and control groups and calculated on the same scale. Alternatively, the standardized mean difference (SMD) was to be calculated using the mean outcome discrepancy between the groups divided by the standard deviation of the outcome among subjects. This method is prioritized when trials utilize differing scales. Both methods were to incorporate a 95% confidence interval (CI) in their analysis. There are unavoidable potential differences between studies, so in this study, we chose a random effects model for data analysis. Based on the Bayesian network framework, according to the PRISMA NMA instructions (23), this study used the Markov chain Monte Carlo method for NMA aggregation and analysis. The network meta-analysis was conducted using the “mvmeta” command in Stata software. Thereafter, the “networkplot” function of Stata was used to generate network plots, which visually demonstrate the layout of various exercise interventions. Indirect and direct comparisons were quantified and validated using the nodal method (24), guided by the instructions outlined in the Stata software. Consistency was confirmed if the p value exceeded 0.05. The results of the network meta-analysis included a network diagram, funnel plot, surface under the cumulative ranking curve, and League table, among others. In the network diagram of PKP/PVP combined with different drug interventions, different nodes represent different interventions. The lines between the two points indicate that there are direct comparisons between the two interventions, and the thickness of the lines reflects the number of studies. The surface area reflects the ranking results of analgesic effects in different regions under the cumulative ranking curve (SUCRA value). The SUCRA value was 0 when the treatment was least effective for analgesia and 1 when the treatment was most effective for analgesia. The relative effectiveness of different treatment options was judged according to the league table generated by NMA analysis. We drew funnel plots of network meta-analyses to assess publication bias.

## 3 Results

### 3.1 General characteristics and characteristics of the included studies

A total of 1,329 documents were obtained through preliminary screening, and two were obtained through manual retrieval. After elimination of duplicate studies, the titles and abstracts of the remaining 272 studies were read. According to the inclusion and exclusion criteria, 18 documents were ultimately included after rescreening (25–42) (Figure 1).

**Figure 2:**
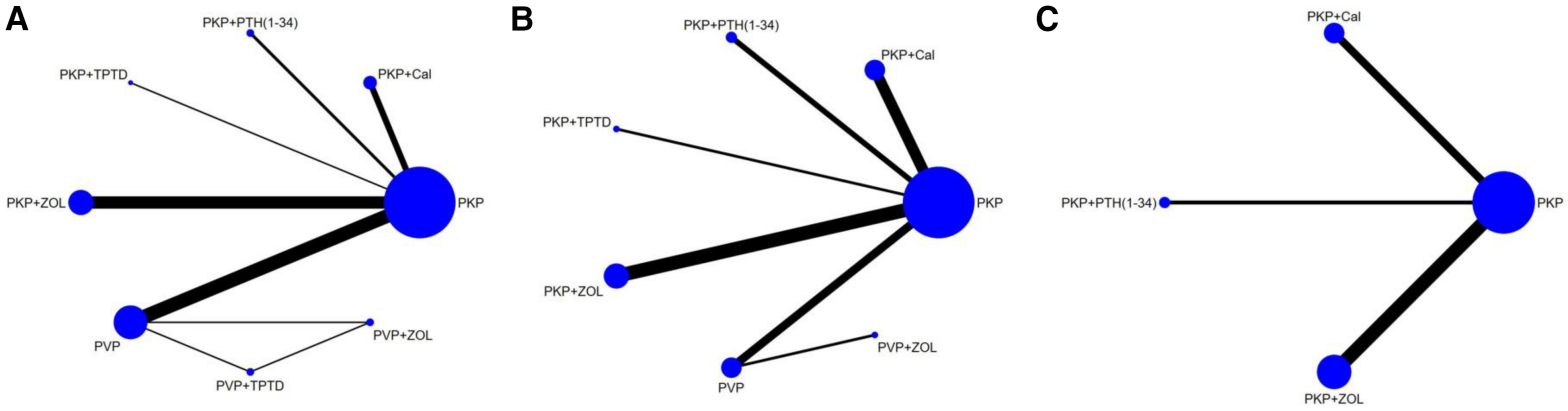
Evidence network of the network meta-analysis. Each node represents the intervention arm; the lines represent direct comparisons in the studies; (**A**) NMA figure for the VAS score; (**B**) NMA figure for ODI; (**C**) NMA figure for BMD; PKP, percutaneous kyphoplasty; PVP, percutaneous vertebroplasty; Cal, calcitonin; PTH 1-34, parathyroid hormone 1-34; TPTD, teriparatide.; ZOL, zoledronic acid.

A total of 18 studies, including 2,374 patients, were included in this network meta-analysis (Table 1). Among them were 12 randomized controlled trials and six other types of studies. In these randomized controlled trials, three drug interventions were included, namely, “zoledronic acid,” “teriparatide (or parathyroid hormone 1-34),” and “calcitonin.” The duration of each study was at least 12 months, and zoledronic acid was the most frequently studied agent (10 trials), followed by teriparatide (5 trials) and calcitonin (4 trials). In this study, both PKP and PVP surgical procedures were compared. To enhance the completeness of the network meta-analysis diagram, nine distinct studies pitting PKP against PVP were incorporated, after a thorough literature search (43–51), in order to highlight the key points, the relevant research information is only included in the Supplementary Materials. (Supplementary Table S5). The primary outcomes of the network meta-analysis were VAS and ODI scores, with 18 and 13 studies reporting results for these two indicators, respectively. The secondary outcome was bone density. The studies of PKP/PVP combined with different drugs that met our inclusion criteria were all from China, and a few studies from other countries were excluded due to short follow-up times (≤6 months). Of the 12 RCTs for which we used the Cochrane risk of bias assessment tool, nine studies were of high quality, whereas 2 studies had a high risk of bias. For the six nonrandomized controlled trials, we used the Newcastle–Ottawa Scale (NOS), for which the average overall quality score was 7 stars. We comprehensively considered the study's design, the measurement of the outcome indicators, and the results of the consistency hypothesis test and conducted this network meta-analysis. The complete NMA map is shown in Figures 2A–C.

### 3.2 Network meta-analysis—primary outcome

#### 3.2.1 Visual analog scale

**Figure 3:**
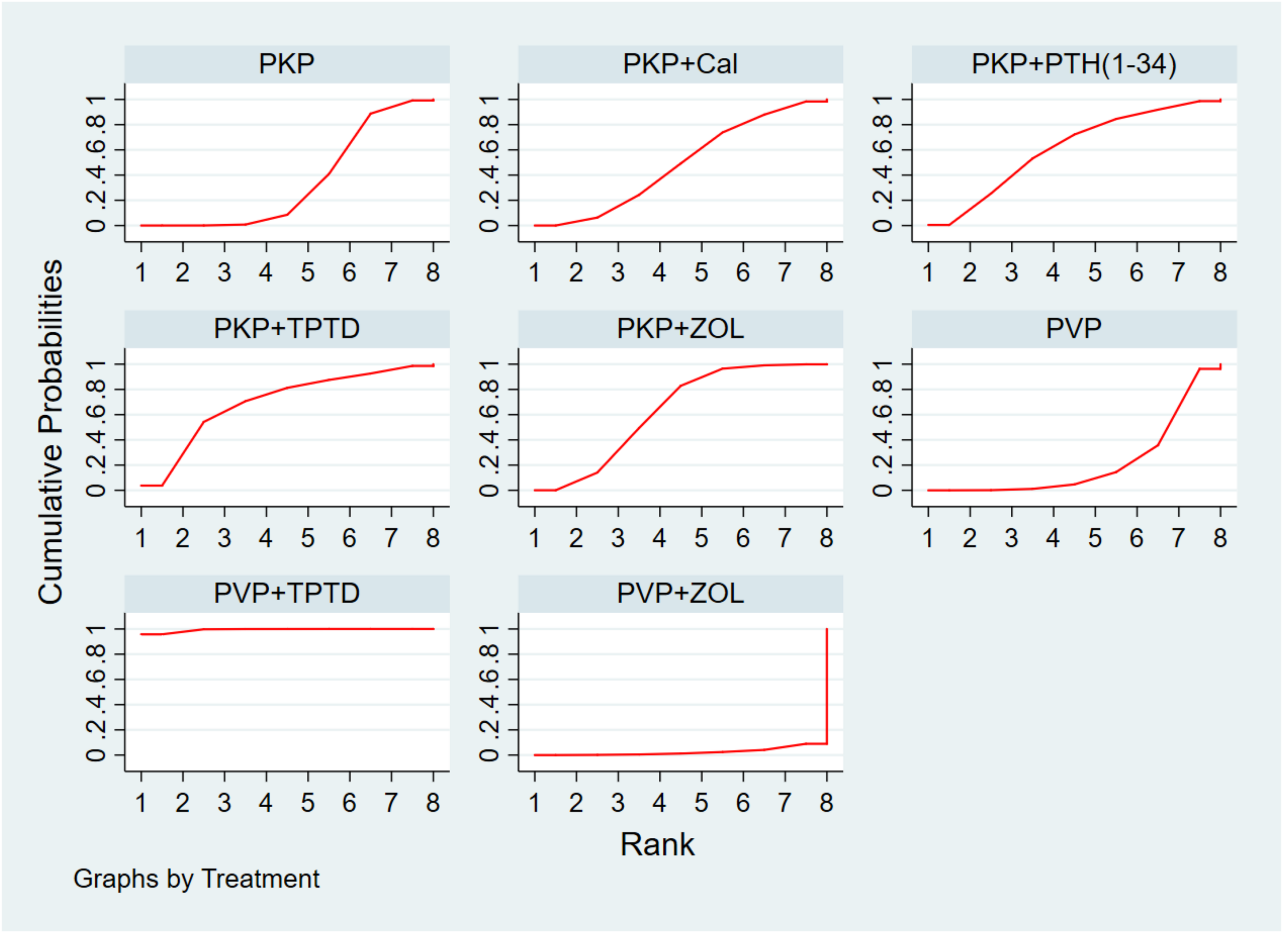
Cumulative probability ranking of VAS score reduction according to treatment regimen. PKP, percutaneous kyphoplasty; PVP, percutaneous vertebroplasty; Cal, calcitonin; PTH 1-34, parathyroid hormone 1-34; TPTD, teriparatide; ZOL, zoledronic acid.

Eighteen studies (1,085 experimental cohorts receiving combined therapy and 1,227 control cohorts) reported feedback from VAS patients treated for 12 months after PKP/PVP. The network meta-analysis revealed that, compared with PVP surgery alone, PVP combined with TPTD was most likely to be the treatment associated with the greatest pain relief [MD = −4.99, 95% CI = (−7.45,−2.52)] (Table 5). According to the SUCRA analysis, the treatment plan involving PVP combined with TPTD was the most common for reducing the VAS score (SUCRA: 99.4%). The second- and third-ranked regimens were PKP combined with TPTD (SUCRA: 69.8%) and PKP combined with ZOL (SUCRA: 63.1%), respectively (Figure 3, Supplementary Table S3A).

**Table 5:** League table on the VAS score.

PVP + ZOL	PVP + TPTD	PVP	PKP + ZOL	PKP + TPTD	PKP + PTH(1-34)	PKP + Cal	PKP
PVP + ZOL	2.27 (−0.18,4.72)	**7.26** **(4.98,9.53)**	**3.23** (**0.09,6.38)**	2.69 (−0.13,5.50)	**3.87** (**0.18,7.56)**	**3.15** (**0.51,5.79)**	1.85 (−0.41,4.11)
−2.27 (−4.72,0.18)	PVP + TPTD	**4.99** (**2.52,7.45)**	0.96 (−1.01,2.94)	0.42 (−0.97,1.80)	1.60 (−1.16,4.36)	0.88 (−0.10,1.86)	−0.42 (−1.37,0.52)
**−7.26** (**−9.53,−4.98)**	**−4.99** (**−7.45,−2.52)**	PVP	**−4.02** (**−7.18,−0.86)**	**−4.57** (**−7.40,−1.74)**	−3.39 (−7.08,0.31)	**−4.11** (**−6.76,−1.45)**	**−5.41** (**−7.69,−3.13)**
**−3.23** (**−6.38,−0.09)**	−0.96 (−2.94,1.01)	**4.02** (**0.86,7.18)**	PKP + ZOL	−0.55 (−2.96,1.87)	0.64 (−2.75,4.03)	−0.08 (−2.29,2.12)	−1.39 (−3.58,0.80)
−2.69 (−5.50,0.13)	−0.42 (−1.80,0.97)	**4.57** (**1.74,7.40)**	0.55 (−1.87,2.96)	PKP + TPTD	1.18 (−1.90,4.27)	0.46 (−1.23,2.16)	−0.84 (−2.52,0.84)
**−3.87** (**−7.56,−0.18)**	−1.60 (−4.36,1.16)	3.39 (−0.31,7.08)	−0.64 (−4.03,2.75)	−1.18 (−4.27,1.90)	PKP + PTH(1-34)	−0.72 (−3.65,2.21)	−2.02 (−4.94,0.89)
**−3.15** (**−5.79,−0.51)**	−0.88 (−1.86,0.10)	**4.11** (**1.45,6.76)**	0.08 (−2.12,2.29)	−0.46 (−2.16,1.23)	0.72 (−2.21,3.65)	PKP + Cal	−1.30 (−2.67,0.06)
−1.85 (−4.11,0.41)	0.42 (−0.52,1.37)	**5.41** (**3.13,7.69)**	1.39 (−0.80,3.58)	0.84 (−0.84,2.52)	2.02 (−0.89,4.94)	1.30 (−0.06,2.67)	PKP

PKP, percutaneous kyphoplasty; PVP, percutaneous vertebroplasty; TPTD, teriparatide; ZOL, zoledronic acid; PTH 1-34, parathyroid hormone 1-34; Cal, calcitonin.

Bold values represents statistical significance *p* < 0.05.

#### 3.2.2 ODI score

**Figure 4:**
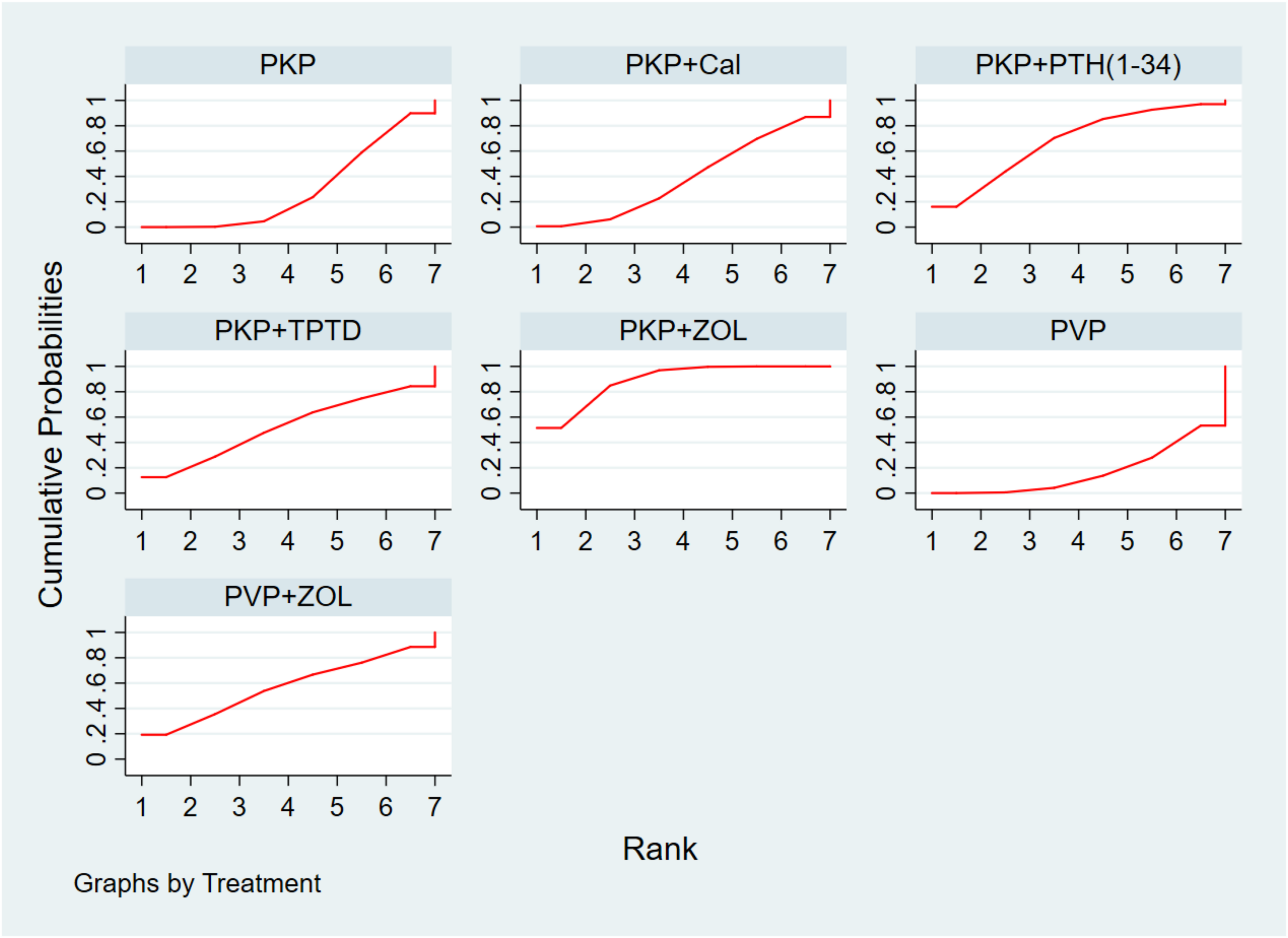
Ranking of the cumulative probability of a reduction in the ODI score according to combination therapy. PKP, percutaneous kyphoplasty; PVP, percutaneous vertebroplasty; Cal, calcitonin; PTH 1-34, parathyroid hormone 1-34; TPTD, teriparatide; ZOL, zoledronic acid.

Thirteen studies (849 experimental and 878 control cohorts who received combined therapy) reported outcomes in patients with ODI who were treated 12 months after PKP/PVP. The results of the network meta-analysis showed that, compared with PKP combined with Cal, PKP combined with ZOL had the highest probability of being the best treatment option for reducing patients’ ODI dysfunction score [MD = −9.11, 95% CI = (−14.27, −3.95)]. Second, the treatment plan for PKP combined with PTH (1-34) was better than that for PKP combined with Cal [MD = −8.04, 95% CI = (−15.79, −0.29)], and there were no significant differences among the other treatment options (Table 6). According to the SUCRA values, PKP combined with ZOL ranked first in terms of the probability of reducing ODI scores with different combined treatment regimens (SUCRA: 88.8%), followed by PKP combined with PTH (1-34) (SUCRA: 67.5%) and PVP combined with ZOL (SUCRA: 56.6%) (Figure 4, Supplementary Table S3B).

**Table 6:** League table on the ODI score.

PVP + ZOL	PVP	PKP + ZOL	PKP + TPTD	PKP + PTH(1-34)	PKP + Cal	PKP
PVP + ZOL	−1.64 (−17.37,14.09)	−5.42 (−13.59,2.74)	−2.42 (−16.46,11.61)	−4.36 (−14.36,5.65)	3.68 (−5.98,13.34)	−7.57 (−18.40,3.27)
1.64 (−14.09,17.37)	PVP	−3.78 (−17.23,9.66)	−0.78 (−18.42,16.86)	−2.72 (−17.35,11.92)	5.32 (−9.08,19.73)	−5.92 (−17.33,5.49)
5.42 (−2.74,13.59)	3.78 (−9.66,17.23)	PKP + ZOL	3.00 (−8.42,14.42)	1.07 (−4.71,6.84)	**9.11** (**3.95,14.27)**	−2.14 (−9.26,4.98)
2.42 (−11.61,16.46)	0.78 (−16.86,18.42)	−3.00 (−14.42,8.42)	PKP + TPTD	−1.93 (−14.73,10.86)	6.11 (−6.42,18.64)	−5.14 (−18.60,8.32)
4.36 (−5.65,14.36)	2.72 (−11.92,17.35)	−1.07 (−6.84,4.71)	1.93 (−10.86,14.73)	PKP + PTH(1-34)	**8.04** (**0.29,15.79)**	−3.21 (−12.38,5.97)
−3.68 (−13.34,5.98)	−5.32 (−19.73,9.08)	**−9.11** (**−14.27,−3.95)**	−6.11 (−18.64,6.42)	**−8.04** (**−15.79,−0.29)**	PKP + Cal	−11.25 (−20.05,2.45)
7.57 (−3.27,18.40)	5.92 (−5.49,17.33)	2.14 (−4.98,9.26)	5.14 (−8.32,18.60)	3.21 (−5.97,12.38)	11.25 (−2.45,20.05)	PKP

PKP, percutaneous kyphoplasty; PVP, percutaneous vertebroplasty; ZOL, zoledronic acid; PTH 1-34, parathyroid hormone 1-34; TPTD, teriparatide; Cal, calcitonin.

Bold values represents statistical significance *p* < 0.05.

#### 3.2.3 Secondary outcome: bone density

**Figure 5:**
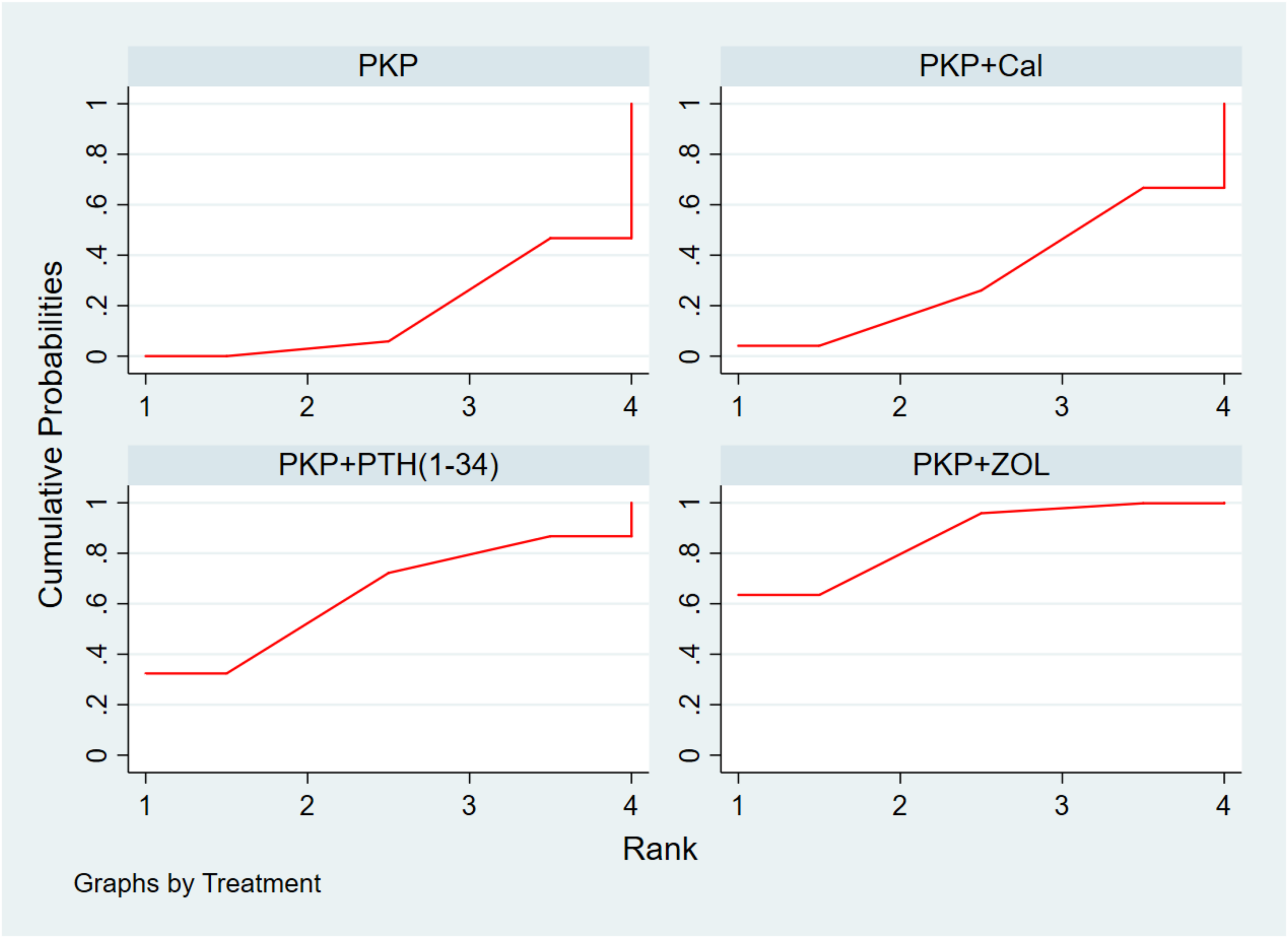
Cumulative probability ranking chart of the protection of BMD by different combination regimens. PKP, percutaneous kyphoplasty; Cal, calcitonin; PTH1-34, parathyroid hormone 1-34; ZOL, zoledronic acid.

Thirteen studies (723 experimental and 747 control cohorts receiving combination therapy) reported feedback outcomes in BMD patients treated for 12 months after PKP. The results of the network meta-analysis showed that, compared with PKP surgery alone, PKP combined with ZOL had the greatest effect on protecting bone mineral density [MD = 0.39, 95% CI = (0.13, 0.65)], but no other treatment plan was significantly different (Table 7). According to the SUCRA, PKP combined with ZOL had the highest correlation with the probability of protecting BMD with different combination therapies (SUCRA: 86.4%), followed by PKP combined with PTH (1-34) (SUCRA: 63.7%). Moreover, for PKP combined with Cal, SUCRA was 32.6% (Figure 5, Supplementary Table S3C).

**Table 7:** League table on BMD.

PKP + ZOL	PKP + PTH(1-34)	PKP + Cal	PKP
PKP + ZOL	−0.13 (−0.70,0.44)	−0.32 (−0.76,0.11)	**−0.39 (−0.65,−0.13)**
0.13 (−0.44,0.70)	PKP + PTH(1-34)	−0.20 (−0.81,0.42)	−0.26 (−0.77,0.25)
0.32 (−0.11,0.76)	0.20 (−0.42,0.81)	PKP + Cal	−0.06 (−0.41,0.29)
**0.39 (0.13,0.65)**	0.26 (−0.25,0.77)	0.06 (−0.29,0.41)	PKP

PKP, percutaneous kyphoplasty; ZOL, zoledronic acid; PTH 1-34, parathyroid hormone 1-34; Cal, calcitonin.

Bold values represents statistical significance *p* < 0.05.

#### 3.2.4 Bias of publication

**Figure 6:**
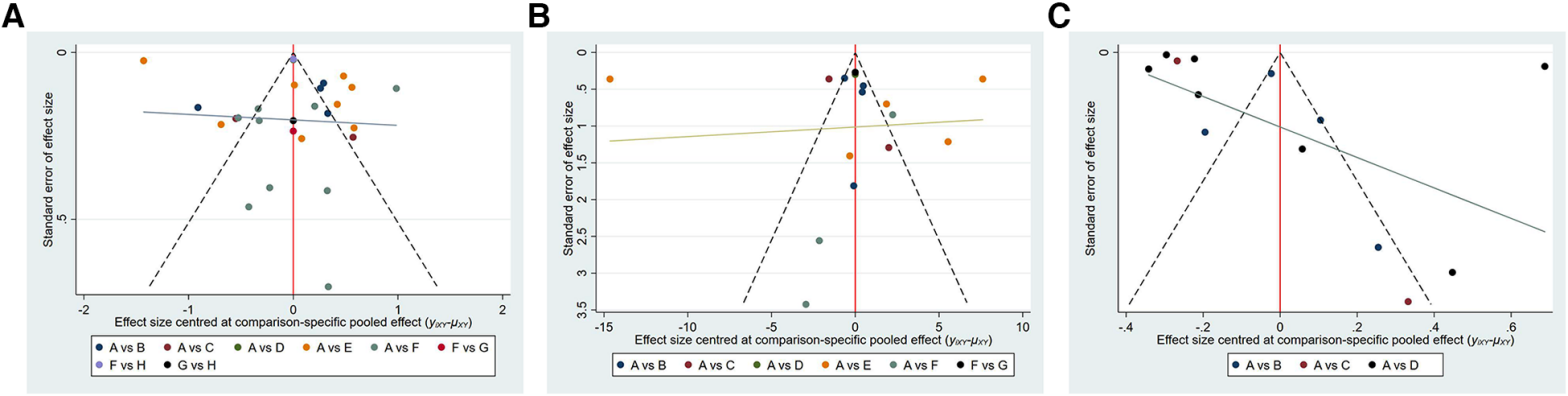
Funnel plot on publication bias. (**A**): VAS; (**B**): ODI; (**C**): BMD. (**A**) VAS: A, percutaneous kyphoplasty; B, Percutaneous kyphoplasty combined with calcitonin;. C, Percutaneous kyphoplasty combined with parathyroid hormone 1-34; D, Percutaneous kyphoplasty combined with teriparatide; E, Percutaneous kyphoplasty combined with zoledronic acid; F, Percutaneous vertebroplasty. G, Percutaneous vertebroplasty combined with teriparatide; H, percutaneous vertebroplasty combined with zoledronic acid; (b) ODI: A-F are the same as in (a); G, percutaneous vertebroplasty combined with zoledronic acid; (c) BMD: (**A–C**) is the same as in (a); D, percutaneous vertebroplasty combined with zoledronic acid.

We used a funnel plot to assess whether there was publication bias in the included studies. The funnel plot revealed no significant publication bias as shown in Figure 6.

## 4 Discussion

Pain is the primary clinical manifestation in OVCF patients and seriously affects their quality of life (52). In recent years, PKP/PVP therapy for OVCFs has been shown not only to quickly relieve pain but also to be associated with less trauma and quick recovery; moreover, PKP/PVP therapy has gradually become a routine method for treating OVCF patients. However, there are several possible complications after surgery, such as new vertebral fractures and postoperative pain; thus, the long-term effect of treatment is uncertain. Previously, several studies based on meta-analysis of PKP/PVP combined with ZOL have shown that PKP/PVP combined with ZOL has a significant effect on relieving pain, reducing the risk of new fractures, and protecting bone mineral density (53–55). However, no studies have compared the efficacy of PKP/PVP combined with other drugs. Therefore, this is the first systematic review and network meta-analysis on the effect of long-term PKP/PVP combined with ZOL, TPTD, or CT in treating postoperative pain in patients with OVCFs.

We included 18 studies, including 2,374 patients, with a modest sample size. Among the treatment options for lowering the VAS score, PVP combined with TPTD may be the most effective treatment for pain relief. TPTD is the 1-34 amino acid fragment of human parathyroid hormone (PTH). It is a synthetic polypeptide hormone that relieves bone pain throughout the body. Specifically, TPTD can stimulate PTH-1 receptor expression and bone formation by regulating the adenylyl cyclase-cyclic adenosine monophosphate-protein kinase A (ATC-A) pathway. It can also reduce the differentiation of stromal cells into adipocytes and increase the number of osteoblasts by inhibiting the transactivation activity of PPAR-γ (56). A recent study revealed that PTH receptors are expressed on sensory nerve cells and that PTH preparations (teriparatide) can first act on neurons to exert analgesic effects before regulating bone metabolism (57). Clinical trials have demonstrated that synthetic metabolic medications, such as teriparatide, can enhance bone density and lower the risk of fractures compared to traditional antiabsorptive drugs. A study involving 428 participants reported that teriparatide can reduce the risk of new vertebral fractures at the 18th month following treatment (58, 59).

According to our study on reducing the ODI dysfunction score, PKP combined with ZOL is the best treatment for relieving low back pain and reducing the degree of dysfunction. Moreover, in the network meta-analysis of BMD, we found that treatment with PKP combined with ZOL had the best therapeutic effect in terms of protecting bone density. As a bisphosphonate with good clinical safety and tolerance, ZOL can specifically bind to bone hydroxyapatite crystals, thereby inhibiting the activity of osteoclasts. Much clinical evidence also shows that ZOL maintains and increases bone density (60). For example, Gnant et al. conducted a study involving 401 premenopausal women to prevent bone loss due to breast cancer treatment. They reported a significant decrease in bone mineral density in the third year in women not taking zoledronic acid. Bisphosphonate drugs, utilized as a primary therapeutic approach, have demonstrated efficacy in mitigating fracture risk and decelerating bone loss. Nonetheless, the effectiveness of these drugs could be subject to genetic variation, leading to differential efficacy across individuals. Such a genetic predisposition might account for the instances of treatment failure and adverse reactions (61). In contrast, bone mineral density remains stable in women treated with zoledronic acid (62, 63). Reid et al. reported that patients who were given a single dose of 5 mg of zoledronic acid had a significantly reduced fracture risk and maintained stable bone mineral density for at least 36 months (64, 65). A 3-year clinical study revealed that annual infusion of ZOL reduced the risk of new osteoporotic fractures and improved patients’ ODI dysfunction scores (41). Multiple lines of clinical evidence also showed that the ODI dysfunction score in the experimental group treated with ZOL was significantly lower than that in the control group (29, 30, 66).

The results of this study showed that, compared with the use of percutaneous vertebroplasty (PVP) alone, the addition of teriparatide helps to reduce VAS pain scores. Compared with percutaneous kyphoplasty (PKP) combined with Cal, the addition of zoledronic acid is a more effective treatment method that can reduce ODI scores and protect bone density. We have drawn valuable conclusions based on good original research; that is, different drugs after PKP/PVP surgery have different effects on reducing VAS and ODI scores and protecting bone density, and these results have practical clinical importance. In future clinical practice, this approach carries substantial implications. By considering different surgical techniques, medical practitioners can select an optimal medication treatment plan for patients, thereby alleviating their pain.

Overall, this study has specific clinical importance. However, there are some notable limitations. First, the number of studies that could be included in the meta-analysis was limited because of the use of different drugs. In addition to RCTs, retrospective or case‒control studies have been performed, which may impact the prediction of the overall results. Despite these shortcomings, summarizing evidence of various levels is a widely accepted strategy. In addition, the sample sizes of several studies were small, and the prediction of treatment efficacy may need to be more accurate. Due to the limitations of the available data, we were unable to incorporate serum biomarkers, such as bone alkaline phosphatase (bALP), the N-terminal propeptide of type I procollagen (PINP), serum crossLaps of type I collagen (bCTx), and urinary crossLaps of type I collagen N-terminal telopeptide (NTx), into our analysis. Nevertheless, these indicators are instrumental in assessing the effectiveness of correlated pharmacological interventions (67). In addition to the pharmaceuticals included in this research, some medications, namely, denosumab, dinosumab, romosozumab, and ibandronate sodium, were not included in our evaluation due to the absence of corresponding pain indices. Future research should further explore the correlation between these drugs and pain (10, 68). Some heterogeneity existed in the studies we included. All of the research underpinning this study originated solely from China. As such, the inclusion of data from additional countries and regions is essential. This is due to the existence of individual variations among patients from different countries, resulting in the application of disparate treatment plans. Consequently, the conclusions drawn from this study may not be generalizable to other regions. We observed heterogeneity in outcomes across the existing body of literature. These discrepancies may stem from the regional discrepancies in the implementation of research within China, and variances in both sample sizes and study intervention measures. Future research, with a broader scale, is necessary for better elucidation of these findings. Therefore, readers should interpret the results of network meta-analyses with caution, and additional in-depth relevant research is needed to confirm these findings in the future.

## 5 Conclusion

This meta-analysis showed that we recommend PVP combined with TPTD to reduce VAS scores. We recommend PKP combined with ZOL to reduce ODI scores and protect bone mineral density. Additionally, since some heterogeneity limits this meta-analysis among published studies, additional high-quality studies are needed to validate our findings.

## Data Availability

The data that support the findings of this study are available from the corresponding author upon reasonable request.

## References

[1] Mullin BH Tickner J Zhu K Kenny J Mullin S Brown SJ Characterisation of genetic regulatory effects for osteoporosis risk variants in human osteoclasts. Genome Biol. (2020) 21:80. 10.1186/s13059-020-01997-2 32216834 PMC7098081

[2] Shim J Lee H Park J Kim H Choi EJ. A non-enzymatic p21 protein inhibitor of stress-activated protein kinases. Nature. (1996) 381:804–6. 10.1038/381804a0 8657286

[3] Fischbacher M Weeks BK Beck BR. The influence of antiresorptive bone medication on the effect of high-intensity resistance and impact training on osteoporotic fracture risk in postmenopausal women with low bone mass: protocol for the MEDEX-OP randomised controlled trial. BMJ Open. (2019) 9:e029895. 10.1136/bmjopen-2019-029895 31492784 PMC6731910

[4] Hu WW He JW Zhang H Wang C Gu JM Yue H No association between polymorphisms and haplotypes of COL1A1 and COL1A2 genes and osteoporotic fracture in postmenopausal Chinese women. Acta Pharmacol Sin. (2011) 32:947–55. 10.1038/aps.2011.37 21602843 PMC4003126

[5] Hu L Sun H Wang H Cai J Tao Y Feng X Cement injection and postoperative vertebral fractures during vertebroplasty. J Orthop Surg Res. (2019) 14:228. 10.1186/s13018-019-1273-z 31324196 PMC6642552

[6] Gou P Zhao Z Yu C Hou X Gao G Zhang T Efficacy of recombinant human parathyroid hormone versus vertebral augmentation procedure on patients with acute osteoporotic vertebral compression fracture. Orthop Surg. (2022) 14:2510–8. 10.1111/os.13470 36017765 PMC9531108

[7] Gu C Huang A Wang Y Liang D Sun P Zhang Z Biomechanics of the unilateral posterosuperior, unipedicular, and bipedicular approaches for treatment by percutaneous vertebroplasty: a comparative study. Am J Transl Res. (2022) 14:3448–55. 35702122 PMC9185039

[8] Scharla S Oertel H Helsberg K Kessler F Langer F Nickelsen T. Skeletal pain in postmenopausal women with osteoporosis: prevalence and course during raloxifene treatment in a prospective observational study of 6 months duration. Curr Med Res Opin. (2006) 22:2393–402. 10.1185/030079906X154097 17257453

[9] Migliorini F Giorgino R Hildebrand F Spiezia F Peretti GM Alessandri-Bonetti M Fragility fractures: risk factors and management in the elderly. Medicina (Kaunas). (2021) 57(10):1119. 10.3390/medicina57101119 PMC8538459 34684156

[10] Migliorini F Maffulli N Colarossi G Eschweiler J Tingart M Betsch M. Effect of drugs on bone mineral density in postmenopausal osteoporosis: a Bayesian network meta-analysis. J Orthop Surg Res. (2021) 16:533. 10.1186/s13018-021-02678-x 34452621 PMC8393477

[11] Ge C Wu X Gao Z Xu Z Hao D Dong L. Comparison of different anesthesia modalities during percutaneous kyphoplasty of osteoporotic vertebral compression fractures. Sci Rep. (2021) 11:11102. 10.1038/s41598-021-90621-9 34045557 PMC8159956

[12] Zhang L Li J Yang H Luo Z Zou J. Histological evaluation of bone biopsy results during PVP or PKP of vertebral compression fractures. Oncol Lett. (2013) 5:135–8. 10.3892/ol.2012.944 23255908 PMC3525491

[13] Wang Y. Application of zoledronic acid in osteoporotic vertebral compression fracture after PKP surgery (master's thesis). The Second Clinical College of Zhengzhou University, Zhengzhou (2020).

[14] Wang B Zhan Y Yan L Hao D. How zoledronic acid improves osteoporosis by acting on osteoclasts. Front Pharmacol. (2022) 13:961941. 10.3389/fphar.2022.961941 36091799 PMC9452720

[15] Chen Y Sun Y. Effect of zoledronic acid on femoral implant subsidence after hip arthroplasty. Chin J Tissue Eng Res. (2022) 26:1812–5. 10.12307/2022.500

[16] Li P Zhao Z Wang L Jin X Shen Y Nan C Minimally effective concentration of zoledronic acid to suppress osteoclasts *in vitro*. Exp Ther Med. (2018) 15:5330–6. 10.3892/etm.2018.6120 29904413 PMC5996712

[17] Huang Y. Combined treatment of vitamin K and teriparatide on bone metabolism and biomechanics in rats with osteoporosis. Exp Ther Med. (2018) 15:315–9. 10.3892/etm.2017.5420 29387190 PMC5768059

[18] Drakopoulos P Flevas DA Galanopoulos IP Lepetsos P Zafeiris C. Off-label use of teriparatide in spine. Cureus. (2021) 13:e16522. 10.7759/cureus.16522 34430132 PMC8376240

[19] Peichl P Holzer LA Maier R Holzer G. Parathyroid hormone 1-84 accelerates fracture-healing in pubic bones of elderly osteoporotic women. J Bone Joint Surg Am. (2011) 93:1583–7. 10.2106/JBJS.J.01379 21915572

[20] Diociaiuti M Polzi LZ Valvo L Malchiodi-Albedi F Bombelli C Gaudiano MC. Calcitonin forms oligomeric pore-like structures in lipid membranes. Biophys J. (2006) 91:2275–81. 10.1529/biophysj.105.079475 16940475 PMC1557561

[21] Luo D Wan X Liu J Tong T. Optimally estimating the sample mean from the sample size, median, mid-range, and/or mid-quartile range. Stat Methods Med Res. (2018) 27:1785–805. 10.1177/0962280216669183 27683581

[22] Wan X Wang W Liu J Tong T. Estimating the sample mean and standard deviation from the sample size, median, range and/or interquartile range. BMC Med Res Methodol. (2014) 14:135. 10.1186/1471-2288-14-135 25524443 PMC4383202

[23] Moher D Shamseer L Clarke M Ghersi D Liberati A Petticrew M Preferred reporting items for systematic review and meta-analysis protocols (PRISMA-P) 2015 statement. Syst Rev. (2015) 4:1. 10.1186/2046-4053-4-1 25554246 PMC4320440

[24] Vats D Flegal JM Jones GL. Multivariate output analysis for Markov chain Monte Carlo. Biometrika. (2019) 106:321–37. 10.1093/biomet/asz002

[25] Su CH Tu PH Yang TC Tseng YY. Comparison of the therapeutic effect of teriparatide with that of combined vertebroplasty with antiresorptive agents for the treatment of new-onset adjacent vertebral compression fracture after percutaneous vertebroplasty. J Spinal Disord Tech. (2013) 26:200–6. 10.1097/BSD.0b013e31823f6298 22134732

[26] Li Y Shu J Wang Z Bi H Guo L He S Effect of teriparatide on residual back pain after percutaneous kyphoplasty for osteoporotic thoracolumbar compression fracture. Chin J Traumatol. (2022) 38:198–204. 10.3760/cma.j.cn501098-20211013-00528

[27] Li Q Chen C Ma X Zhang H Feng H. Parathyroid hormone 1-34 in the treatment of adjacent vertebral refracture after percutaneous kypho-plasty for thoracolumbar osteoporotic compression fracture. Chin J Ortho Traumatol. (2020) 22:355–9. 10.3760/cma.j.cn115530-20191022-00363

[28] Yuan J Zhang Y Sun L Miu L Huo J Ma X Clinical effects of parathyroid hormone 1-34 combined with percutaneous kyphoplasty on osteoporotic vertebral compression fracture. Chin J Osteo Bone Min Res. (2017) 10:34–42. 10.3969/j.issn.1674-2591.2017.01.007

[29] Liu B Gan F Ge Y Yu H. Clinical efficacy analysis of percutaneous kyphoplasty combined with zoledronic acid in the treatment and prevention of osteoporotic vertebral compression fractures. J Invest Surg. (2018) 31:425–30. 10.1080/08941939.2017.1339151 28829670

[30] Shi C Zhang M Cheng AY Huang ZF. Percutaneous kyphoplasty combined with zoledronic acid infusion in the treatment of osteoporotic thoracolumbar fractures in the elderly. Clin Interv Aging. (2018) 13:853–61. 10.2147/CIA.S146871 29765210 PMC5942393

[31] Huang ZF Xiao SX Liu K Xiong W. Effectiveness analysis of percutaneous kyphoplasty combined with zoledronic acid in treatment of primary osteoporotic vertebral compression fractures. Pain Physician. (2019) 22:63–8. 10.36076/ppj/2019.22.63 30700069

[32] Zhang J Zhang T Xu X Cai Q Zhao D. Zoledronic acid combined with percutaneous kyphoplasty in the treatment of osteoporotic compression fracture in a single T12 or L1 vertebral body in postmenopausal women. Osteoporos Int. (2019) 30:1475–80. 10.1007/s00198-019-04896-w 30976888

[33] Hu W Wang H Shi X Song Y Zhang G Xing S Effect of preoperative zoledronic acid administration on pain intensity after percutaneous vertebroplasty for osteoporotic vertebral compression fractures. Pain Res Manag. (2020) 2020:8039671. 10.1155/2020/8039671 32831984 PMC7421713

[34] Liu K Tan G Sun W Lu Q Tang J Yu D. Percutaneous kyphoplasty combined with zoledronic acid for the treatment of primary osteoporotic vertebral compression fracture: a prospective, multicenter study. Arch Orthop Trauma Surg. (2023) 143:3699–706. 10.1007/s00402-022-04557-4 35933563

[35] Zhang J Tan L Liu M Zhao T Qi B. The effect of zoledronic acid combined with PKP in the treatment of osteoporotic vertebral body compression fractures. Int J Clin Exp Med. (2020) 13:9073–9.

[36] Dang X Guo C Chen Z Zhang D Wu R. Efficacy of PKP combined with salmon calcitonin in the treatment of elderly patients with osteoporotic thoracolumbar compression fractures. J Cervic Lumbody. (2019) 40:555–6. 10.3969/j.issn.1005-7234.2019.04.043

[37] Hao J. The short-term efficacy analysis of percutaneous kyphoplasty combined with salmon calcitonin in the treatment of osteoporotic vertebral compression fractures (master's thesis). Dalian Medical University, Dalian, Liaoning (2018).

[38] Zhong F. Percutaneous vertebral plasty combined salmon calcitonin treatment of senile osteoporotic thoracolumbar compression fractures of the clinical curative effect. Chin J Clin Ratio Drug Use. (2021) 14:99–101. 10.15887/j.cnki.13-1389/r.2021.06.038

[39] Wang X Xu B Ye X Yang Y Wang G. Effects of different treatments on patients with osteoporotic fracture after percutaneous kyphoplasty. Chin J Orthop Traumatol. (2015) 28:512–6. 10.3969/j.issn.1003-0034.2015.06.007 26255474

[40] Yi H Chen T Gan J Dong Z Liu D Zheng Y Effects of percutaneous kyphoplasty combined with zoledronic acid injection on osteoporotic vertebral compression fracture and bone metabolism indices. J Neurosurg Sci. (2024) 68:80–8. 10.23736/S0390-5616.20.05117-6 33297608

[41] Lu K Yin Y Li C Jin Y Shan HQ. Efficacy of annual zoledronic acid in initial percutaneous kyphoplasty patients with osteoporotic vertebral compression fractures: a 3-year follow-up study. Osteoporos Int. (2021) 32:1429–39. 10.1007/s00198-020-05816-z 33462653

[42] Hao M Li J Jin C Luan X Luan J Li Q Clinical effect of teriparatide in preventing recurrent fracture after percutaneous vertebroplasty for osteoporotic vertebral compression fracture. J Prec Med. (2021) 36:399–403. 10.13362/j.jpmed.202105006

[43] Deng L Lv N Hu X Guan Y Hua X Pan Z Comparison of efficacy of percutaneous vertebroplasty versus percutaneous kyphoplasty in the treatment of osteoporotic vertebral asymmetric compression fracture. World Neurosurg. (2022) 167:e1225–30. 10.1016/j.wneu.2022.09.017 36089275

[44] Wu Y Wang F Zhou J Liu C Wu R. Analysis of clinical effects of percutaneous vertebroplasty and percutaneous kyphoplasty in treating osteoporotic vertebral compression fracture. Chin J Orthop Traumatol. (2014) 27:385–9. 10.3969/j.issn.1003-0034.2014.05.008 25167667

[45] Du J Li X Lin X. Kyphoplasty versus vertebroplasty in the treatment of painful osteoporotic vertebral compression fractures: two-year follow-up in a prospective controlled study. Acta Orthop Belg. (2014) 80:477–86. 26280719

[46] Hu C-H Li Q-P Wang C Liu Q-P Long H-G. Analysis of clinical effects of three operative methods for osteoporotic vertebral compression fracture. Chin J Ortho Traumatol. (2016) 29:619–24. 10.3969/j.issn.1003-0034.2016.07.007 29232779

[47] Zhou J-L Liu S-Q Ming J-H Peng H Qiu B. Comparison of therapeutic effect between percutaneous vertebroplasty and kyphoplasty on vertebral compression fracture. Chin J Traumatol. (2008) 11:42–4. 10.1016/S1008-1275(08)60009-7 18230291

[48] Yan D Duan L Li J Soo C Zhu H Zhang Z. Comparative study of percutaneous vertebroplasty and kyphoplasty in the treatment of osteoporotic vertebral compression fractures. Arch Orthop Trauma Surg. (2011) 131:645–50. 10.1007/s00402-010-1188-y 20848113

[49] Schofer MD Efe T Timmesfeld N Kortmann H-R Quante M. Comparison of kyphoplasty and vertebroplasty in the treatment of fresh vertebral compression fractures. Arch Orthop Trauma Surg. (2009) 129:1391–9. 10.1007/s00402-009-0901-1 19471946

[50] Röllinghoff M Siewe J Zarghooni K Sobottke R Alparslan Y Eysel P Effectiveness, security and height restoration on fresh compression fractures–a comparative prospective study of vertebroplasty and kyphoplasty. Min-Minimally Inv Neurosurg. (2009) 52:233–7. 10.1055/s-0029-1243631 20077364

[51] Folman Y Shabat S. A comparison of two new technologies for percutaneous vertebral augmentation: confidence vertebroplasty vs. sky kyphoplasty. Sat. (2011) 8:23. 21838179

[52] Long Y Yi W Yang D. Advances in vertebral augmentation systems for osteoporotic vertebral compression fractures. Pain Res Manag. (2020) 2020:3947368. 10.1155/2020/3947368 33376566 PMC7738798

[53] Sun Y Ma H Yang F Tang X Yi P Tan M. Clinical efficacy and safety of zoledronic acid combined with PVP/PKP in the treatment of osteoporotic vertebral compression fracture: a systematic review and meta-analysis of randomized controlled trials. Biomed Res Int. (2021) 2021:6650358. 10.1155/2021/6650358 33928158 PMC8049795

[54] Zhuang M Cai B Wang F. Effectiveness and safety of percutaneous kyphoplasty combined with zoledronic acid in treatment of osteoporotic vertebral compression fractures: a meta-analysis. Arch Orthop Trauma Surg. (2022) 142:2435–43. 10.1007/s00402-021-03858-4 33713186

[55] Wu XG Zhang DY Zhu BQ Li AM. Efficacy of zoledronic acid with percutaneous kyphoplasty/vertebroplasty in the treatment of osteoporotic vertebral compression fractures: a systematic review and meta-analysis. Eur Rev Med Pharmacol Sci. (2020) 24:12358–67. 10.26355/eurrev_202012_24030 33336756

[56] Shenghan L Licheng Z Peifu T. Advances in teriparatide-based treatment of osteoporotic fractures. Acad J Chin Pla Med School. (2016) 37:522–4. 10.3969/j.issn.2095-5227.2016.05.029

[57] Tanaka T Takao-Kawabata R Takakura A Shimazu Y Nakatsugawa M Ito A Teriparatide relieves ovariectomy-induced hyperalgesia in rats, suggesting the involvement of functional regulation in primary sensory neurons by PTH-mediated signaling. Sci Rep. (2020) 10:5346. 10.1038/s41598-020-62045-4 32210273 PMC7093455

[58] Saag KG Shane E Boonen S Marin F Donley DW Taylor KA Teriparatide or alendronate in glucocorticoid-induced osteoporosis. N Engl J Med. (2007) 357:2028–39. 10.1056/NEJMoa071408 18003959

[59] Migliorini F Colarossi G Eschweiler J Oliva F Driessen A Maffulli N. Antiresorptive treatments for corticosteroid-induced osteoporosis: a Bayesian network meta-analysis. Br Med Bull. (2022) 143:46–56. 10.1093/bmb/ldac017 35641234 PMC9494254

[60] Liu M Guo L Pei Y Li N Jin M Ma L Efficacy of zoledronic acid in treatment of osteoporosis in men and women-a meta-analysis. Int J Clin Exp Med. (2015) 8:3855–61. 26064284 PMC4443118

[61] Conti V Russomanno G Corbi G Toro G Simeon V Filippelli W A polymorphism at the translation start site of the vitamin D receptor gene is associated with the response to anti-osteoporotic therapy in postmenopausal women from southern Italy. Int J Mol Sci. (2015) 16:5452–66. 10.3390/ijms16035452 25764158 PMC4394486

[62] Gnant MF Mlineritsch B Luschin-Ebengreuth G Grampp S Kaessmann H Schmid M Zoledronic acid prevents cancer treatment-induced bone loss in premenopausal women receiving adjuvant endocrine therapy for hormone-responsive breast cancer: a report from the Austrian breast and colorectal cancer study group. J Clin Oncol. (2007) 25:820–8. 10.1200/JCO.2005.02.7102 17159195

[63] Hines SL Mincey BA Sloan JA Thomas SP Chottiner E Loprinzi CL Phase III randomized, placebo-controlled, double-blind trial of risedronate for the prevention of bone loss in premenopausal women undergoing chemotherapy for primary breast cancer. J Clin Oncol. (2009) 27:1047–53. 10.1200/JCO.2008.19.1783 19075260 PMC2667810

[64] Reid IR Black DM Eastell R Bucci-Rechtweg C Su G Hue TF Reduction in the risk of clinical fractures after a single dose of zoledronic acid 5 milligrams. J Clin Endocrinol Metab. (2013) 98:557–63. 10.1210/jc.2012-2868 23293335

[65] Shieh A Greendale GA Cauley JA Karlamangla AS. The association between fast increase in bone turnover during the menopause transition and subsequent fracture. J Clin Endocrinol Metab. (2020) 105:e1440–8. 10.1210/clinem/dgz281 31840764 PMC7067542

[66] Huang S Zhu X Xiao D Zhuang J Liang G Liang C Therapeutic effect of percutaneous kyphoplasty combined with anti-osteoporosis drug on postmenopausal women with osteoporotic vertebral compression fracture and analysis of postoperative bone cement leakage risk factors: a retrospective cohort study. J Orthop Surg Res. (2019) 14:452. 10.1186/s13018-019-1499-9 31852483 PMC6921385

[67] Migliorini F Maffulli N Spiezia F Peretti GM Tingart M Giorgino R. Potential of biomarkers during pharmacological therapy setting for postmenopausal osteoporosis: a systematic review. J Orthop Surg Res. (2021) 16:351. 10.1186/s13018-021-02497-0 34059108 PMC8165809

[68] Migliorini F Colarossi G Baroncini A Eschweiler J Tingart M Maffulli N. Pharmacological management of postmenopausal osteoporosis: a level I evidence based - expert opinion. Expert Rev Clin Pharmacol. (2021) 14:105–19. 10.1080/17512433.2021.1851192 33183112

