# Supplementary material for: Effects of different drugs in combination with PKP/PVP on postoperative pain in patients with osteoporotic compression fractures: a network meta-analysis
Source: Front Surg. 2024 Jul 23;11:1349351. doi: 10.3389/fsurg.2024.1349351 (PMC11302139; doi:10.3389/fsurg.2024.1349351)
Supplement: Supplementary file 1 [file Table1.docx]

Supplementary Material

**Table S1. Quality Assessment of 12 Studies on the** **Cochrane Collaboration Risk of Bias**

| Study | Random  Sequence  generation | Allocation  concealment | Blinding of  participants  and personnel | Blinding of  outcome  assessment | Incomplete  outcome data | Selective  reporting | Other bias | Overall Score  (Category) |
| --- | --- | --- | --- | --- | --- | --- | --- | --- |
| Su | Unclear; | low; | low; | low; | low; | Unclear; | low; | 2 |
| Yuan | Low; | unclear; | unclear; | low; | low; | low; | low; | 2 |
| Liu | Low; | low; | unclear; | low; | low; | low; | low; | 1 |
| Huang | Low; | unclear; | unclear; | low; | unclear; | low; | high; | 4 |
| Zhang | Low; | low; | high | unclear; | low; | low; | low; | 2 |
| Hu | Low; | unclear; | unclear; | low; | low; | low; | low; | 2 |
| Liu | Low; | low; | unclear; | low; | low; | low; | low; | 1 |
| Zhang | Low; | unclear; | unclear; | unclear; | low; | low; | low; | 3 |
| Zhong | Low; | unclear; | unclear; | unclear; | low; | low; | unclear; | 4 |
| Wang | Low; | low; | low; | low; | low; | low; | unclear; | 1 |
| Yi | Low; | low; | low; | unclear; | low; | low; | low; | 1 |
| Lu | Low; | low; | low; | low; | low; | low; | unclear; | 1 |

**Table S2. Quality Assessment of 6 Studies on the Newcastle-Ottawa Scale**

| Study | Adequate definition of cases | Represent-  ativeness of the cases | Selection of controls | Definition of controls | Comparability  Control for important factor* | Ascertain- ment of exposure | Same method of ascertainment for cases and controls | Nonresponse rate | Overall Score  (Category) |
| --- | --- | --- | --- | --- | --- | --- | --- | --- | --- |
| Li a | ★ | ★ | ☆ | ☆ | ★☆ | ★ | ★ | ★ | 6 |
| Li b | ★ | ★ | ★ | ☆ | ★☆ | ★ | ★ | ★ | 7 |
| Shi | ★ | ★ | ★ | ★ | ★★ | ★ | ★ | ★ | 9 |
| Hao a | ★ | ★ | ☆ | ★ | ★☆ | ☆ | ★ | ★ | 6 |
| Dang | ★ | ★ | ☆ | ☆ | ★☆ | ★ | ★ | ★ | 6 |
| Hao b | ★ | ★ | ☆ | ☆ | ★☆ | ★ | ★ | ★ | 6 |

* Medication was an important confounding factor.

**Table S3. Treatment Relative Ranking of Estimated probabilities**

**Table S3A. VAS**

| Treatment | SUCRA | Pr Best | Mean Rank |
| --- | --- | --- | --- |
| PKP | 34 | 0 | 5.6 |
| PKP+Cal | 48.6 | 0 | 4.6 |
| PTH (1-34) | 60.9 | 0.4 | 3.7 |
| PKP+TPTD | 69.8 | 3.7 | 3.1 |
| PKP+ZOL | 63.1 | 0.1 | 3.6 |
| PVP | 21.8 | 0 | 6.5 |
| PVP+TPTD | 99.4 | 95.8 | 1 |
| PVP+ZOL | 2.5 | 0 | 7.8 |

**Table S3B. ODI**

| Treatment | SUCRA | Pr Best | Mean Rank |
| --- | --- | --- | --- |
| PKP | 29.5 | 0 | 5.2 |
| PKP+Cal | 38.9 | 0.7 | 4.7 |
| PTH (1-34) | 67.5 | 16.1 | 2.9 |
| PKP+TPTD | 52 | 12.6 | 3.9 |
| PKP+ZOL | 88.8 | 51.4 | 1.7 |
| PVP | 16.6 | 0 | 6 |
| PVP+ZOL | 56.6 | 19.3 | 3.6 |

**Table S3C. BMD**

| Treatment | SUCRA | Pr Best | Mean Rank |
| --- | --- | --- | --- |
| PKP | 17.5 | 0 | 3.5 |
| PKP+Cal | 32.6 | 4.3 | 3.0 |
| PTH(1-34) | 63.7 | 31.4 | 2.1 |
| PKP+ZOL | 86.4 | 64.3 | 1.4 |

Abbreviation: VAS, Visual Analogue Scale. ODI, Oswestry Disability Index. BMD, Bone Mineral Density. PKP, percutaneous kyphoplasty. PVP, percutaneous vertebro plasty. Cal, calcitonin. PTH 1-34, parathyroid hormone 1-34. TPTD, teriparatide. ZOL, zoledronic acid. SUCRA, surface under the cumulative ranking area.

**Table S4. Treatment Relative Ranking of Predictive probabilities**

**Table S4A. VAS**

| Treatment | SUCRA | Pr Best | Mean Rank |
| --- | --- | --- | --- |
| PKP | 39.7 | 0.9 | 5.2 |
| PKP+Cal | 47.1 | 2.3 | 4.7 |
| PTH(1-34) | 56.1 | 6.3 | 4.1 |
| PKP+TPTD | 63.1 | 13.7 | 3.6 |
| PKP+ZOL | 54.8 | 3.2 | 4.2 |
| PVP | 33.4 | 0.2 | 5.7 |
| PVP+TPTD | 92.4 | 73.4 | 1.5 |
| PVP+ZOL | 13.5 | 0 | 7.1 |

**Table S4B ODI**

| Treatment | SUCRA | Pr Best | Mean Rank |
| --- | --- | --- | --- |
| PKP | 38.5 | 1.8 | 4.7 |
| PKP+Cal | 44.1 | 4.6 | 4.4 |
| PTH(1-34) | 64.6 | 20 | 3.1 |
| PKP+TPTD | 53.4 | 15.2 | 3.8 |
| PKP+ZOL | 81.2 | 41.7 | 2.1 |
| PVP | 20.9 | 1.3 | 5.7 |
| PVP+ZOL | 47.3 | 15.4 | 4.2 |

**Table S4C. BMD**

| Treatment | SUCRA | Pr Best | Mean Rank |
| --- | --- | --- | --- |
| PKP | 28.6 | 5.6 | 3.1 |
| PKP+Cal | 37.4 | 12.4 | 2.9 |
| PTH(1-34) | 58.8 | 32.1 | 2.2 |
| PKP+ZOL | 75.2 | 49.9 | 1.7 |

Abbreviation: VAS, Visual Analogue Scale. ODI, Oswestry Disability Index. BMD, Bone Mineral Density. PKP, percutaneous kyphoplasty. PVP, percutaneous vertebro plasty. Cal, calcitonin. PTH 1-34, parathyroid hormone 1-34. TPTD, teriparatide. ZOL, zoledronic acid. SUCRA, surface under the cumulative ranking area.

**Table S5. Characteristics of Studies Focused Solely on PKP/PVP Included in the Network Meta-Analysis**

| Study | Study design | Country | No. of patients | | Age | | Gender (Male) | |
| --- | --- | --- | --- | --- | --- | --- | --- | --- |
|  |  |  | PKP | PVP | PKP | PVP | PKP | PVP |
| Wu Yao 2014 [1] | Retrospective comparative study | China | 20 | 20 | 65.12 | 66.37 | 9 | 12 |
| Du Junhua 2014 [2] | Prospective comparative study | China | 44 | 42 | 75.6 | 72.1 | 8 | 9 |
| Hu Chunhua 2016 [3] | Retrospective cohort | China | 30 | 30 | 67.44 | 68.73 | 18 | 18 |
| Zhou Jianlin 2008 [4] | Retrospective cohort | China | 42 | 56 | 64 | 62 | 17 | 21 |
| Denglu Yan 2011 [5] | Retrospective cohort | China | 98 | 94 | 76.9 | 77.2 | 41 | 39 |
| Markus Dietmar Schofer 2009 [6] | Prospective comparative study | Germany | 30 | 30 | 72.5 | 73.8 | 8 | 6 |
| M. Rollinghoff 2009 [7] | Prospective comparative study | USA | 53 | 52 | 68.9 | | 20 | |
| Yoram Folman 2011 [8] | Prospective cohort | Israel | 31 | 14 | 70.74 | 75.57 | 9 | 5 |

**References**

[1] Y. Wu, F. Wang, J. Zhou, C. Liu, and R. Wu, Analysis of clinical effects of percutaneous vertebroplasty and percutaneous kyphoplasty in treating osteoporotic vertebral compression fracture. China Journal of Orthopaedics and Traumatology 27 (2014) 385-389.

[2] J. Du, X. Li, and X. Lin, Kyphoplasty versus vertebroplasty in the treatment of painful osteoporotic vertebral compression fractures: two-year follow-up in a prospective controlled study. Acta Orthop Belg 80 (2014) 477-486.

[3] C.-H. Hu, Q.-P. Li, C. Wang, Q.-P. Liu, and H.-G. Long, Analysis of clinical effects of three operative methods for osteoporotic vertebral compression fracture. Zhongguo Gu Shang= China journal of orthopaedics and traumatology 29 (2016) 619-624.

[4] J.-l. Zhou, S.-q. Liu, J.-h. Ming, H. Peng, and B. Qiu, Comparison of therapeutic effect between percutaneous vertebroplasty and kyphoplasty on vertebral compression fracture. Chinese Journal of Traumatology 11 (2008) 42-44.

[5] D. Yan, L. Duan, J. Li, C. Soo, H. Zhu, and Z. Zhang, Comparative study of percutaneous vertebroplasty and kyphoplasty in the treatment of osteoporotic vertebral compression fractures. Archives of orthopaedic and trauma surgery 131 (2011) 645-650.

[6] M.D. Schofer, T. Efe, N. Timmesfeld, H.-R. Kortmann, and M. Quante, Comparison of kyphoplasty and vertebroplasty in the treatment of fresh vertebral compression fractures. Archives of orthopaedic and trauma surgery 129 (2009) 1391-1399.

[7] M. Röllinghoff, J. Siewe, K. Zarghooni, R. Sobottke, Y. Alparslan, P. Eysel, and K.-S. Delank, Effectiveness, security and height restoration on fresh compression fractures–a comparative prospective study of vertebroplasty and kyphoplasty. min-Minimally Invasive Neurosurgery 52 (2009) 233-237.

[8] Y. Folman, and S. Shabat, A comparison of two new technologies for percutaneous vertebral augmentation: confidence vertebroplasty vs. sky kyphoplasty. Sat 8 (2011) 23.
